# Liver pro-oncogenic potential of SERPINB3

**DOI:** 10.18632/oncoscience.76

**Published:** 2014-09-03

**Authors:** Patrizia Pontisso, Andrea Martini, Cristian Turato

**Affiliations:** Department of Medicine, University of Padua (Italy)

Hepatocellular Carcinoma (HCC) is a growing sanitary problem worldwide. This tumor is indeed the third most deadly and the fifth most common cancer all over the word, with an incidence that, at least in the United States, has doubled in the past two decades [1].

Current studies suggest that HCC can derive from liver progenitor cells or cancer stem cells (CSCs) in which signaling pathway similar to those identified in liver cancer (e.g., Wnt, TGF-β, Notch, Hedgehog, and PI3K/AKT/mTOR), are activated. These findings support the hypothesis that CSCs contribute to the molecular heterogeneity and chemoresistance of this tumor [2]. In addition, the progenitor-like subtype of hepatocellular carcinoma is associated with a poor prognosis [2].

Cancer stem cells are neoplastic cells that possess distinct survival mechanisms and stem cell properties responsible for tumor formation and progression. Although the origin of CSCs in liver cancer is not fully elucidated, has been recently demonstrated that any hepatic lineage cell (tumor progenitor cells, stem cells, or dedifferentiated cells) can be reprogrammed into CSC by activating diverse cell type-specific pathways [3].

The role of preneoplastic lesions in HCC remains still controversial. A recent study [4] has demonstrated that in foci of altered hepatocytes (FAH), consisting in highly proliferative cells, compared to the parenchyma of cirrhotic liver, resides aggregates of normal hepatocytes, oval cells and progenitor cells with high expression of specific markers, including CD90, CD44, EpCAM and alpha-fetoprotein (AFP). It was further demonstrated that this subset of cells with stem/progenitor are tumorigenic in damaged livers (i.e., in chronic injury and compensatory proliferation) and in this context the microenvironment, IL-6 and TGF-β signaling are required to promote *in vivo* growth and malignant progression of hepatic cancer progenitor cells and tumor metastasis [4].

In the liver, the ov-serpin SERPINB3 (or SCCA1) and its isoforms SERPINB4 (or SCCA2) are undetectable in normal hepatocytes, but their expression progressively increases in chronic liver diseases, dysplastic nodules and HCC, suggesting that they may be involved in relatively early events of hepatocarcinogenesis, although their specific role has not been defined yet [5].

In the article “SERPINB3 is associated with TGF-β1 and cytoplasmic β-catenin expression in hepatocellular carcinomas with poor prognosis” [5] Turato et al, for the first time, provided evidence that in liver tumors the expression of the protease inhibitor SERPINB3 was correlated with the molecular markers of poor prognosis, (TGF-β signalling associated with Wnt target gene expression). High expression of SERPINB3 was indeed significantly associated with early tumor recurrence, which is commonly regarded as a true metastasis, due to dissemination of primary tumor cells then representing a subset of most aggressive HCCs. These results are in agreement with previous studies *in vitro*, demonstrating that SERPINB3 can enhance TGF-β expression and induce epithelial-mesenchymal transition, with increased invasiveness and cell proliferation [5].

**Figure 1:**
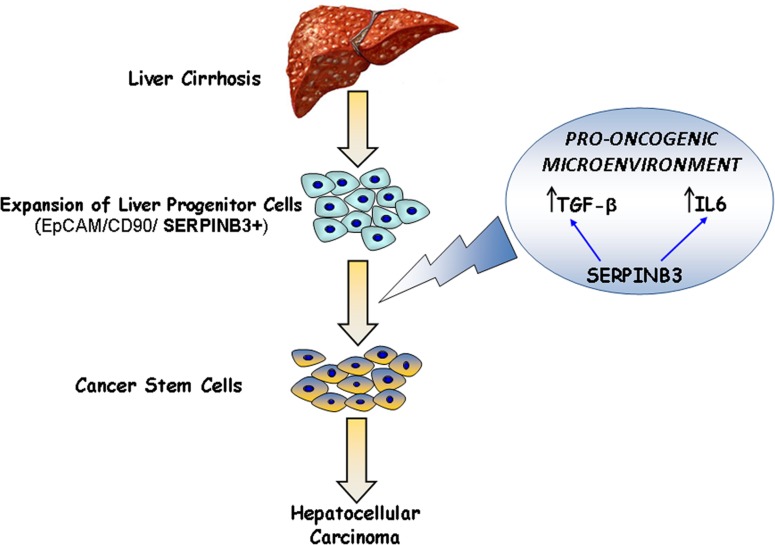
Proposed involvement of SERPINB3 in liver carcinogenesis The presence of this serpin in liver progenitor cells and its contribution to the increase of microenvironment cytokines (LI-6, TGF-β) might be crucial steps in liver carcinogenesis.

The presence of SERPINB3 in human progenitor cells compartment has been recently demonstrated by our group. This serpin was indeed detected in EpCAM + cell fractions sorted from human foetal and adult livers and in the same ductular structures that express stem/progenitor cell markers. Moreover, the mouse homologous, Serpinb3b, was induced in a mouse model of liver stem/progenitor cell activation [6].

Expression of SERPINB3 protein was also observed in hepatoblastoma, considered an embryonal tumour of the liver, especially in tumors with more advanced stage. The intensity of the protein signal was higher in less differentiated components of the tumor and in the clusters of undifferentiated variant, named small undifferentiated cell (SCUD) pattern, considered as primitive uncommitted progenitor cells [7].

Recent findings revealed that SERPINB3/4 isoforms are RAS-responsive factors that can lead to inflammatory protein production and tumorigenesis. They are transcriptionally upregulated via MAPK and the ETS family transcription factors PEA3. The increased expression of these serpin family members determines NF-kB activation, essential for RAS-mediated cytokine production and tumor growth. The positive correlation between Ras mutation, enhanced SERPINB3 expression and IL-6 expression was documented in human colorectal and pancreatic tumour samples, reflecting the inflammatory and protumorigenic role of this serpin [8].

These last results, taken together with the finding that high SERPINB3 expression was detected in liver progenitor cells and in the most aggressive liver tumors [5,7] suggest that this serpin, may determine a pro-tumorogenic microenvironment milieu and cellular features with a high pro-oncogenic potential.
